# Neural differences between chromatic- and luminance-driven attentional salience in visual search

**DOI:** 10.1167/jovi.20.3.5

**Published:** 2020-03-20

**Authors:** Amanda Hardman, Thomas Töllner, Jasna Martinovic

**Affiliations:** School of Psychology, University of Aberdeen, Aberdeen, UK; Department of Experimental Psychology, Ludwig-Maximilians-University Munich, Munich, Germany; School of Psychology, University of Aberdeen, Aberdeen, UK

**Keywords:** attention, color, luminance, EEG, ERP, visual search, salience, cone-opponent mechanisms, posterior contralateral negativity

## Abstract

Previous electroencephalographic research on attentional salience did not fully capture the complexities of low-level vision, which relies on both cone-opponent chromatic and cone-additive luminance mechanisms. We systematically varied color and luminance contrast using a visual search task for a higher contrast target to assess the degree to which the salience-computing attentional mechanisms are constrained by low-level visual inputs. In our first experiment, stimuli were defined by contrast that isolated chromatic or luminance mechanisms. In our second experiment, targets were defined by contrasts that isolated or combined achromatic and chromatic mechanisms. In both experiments, event-related potential waveforms contralateral and ipsilateral to the target were qualitatively different for chromatic- compared to luminance-defined stimuli. The same was true of the difference waves computed from these waveforms, with isoluminant stimuli eliciting a mid-latency posterior contralateral negativity (PCN) component and achromatic stimuli eliciting a complex of multiple components, including an early posterior contralateral positivity followed by a late-latency PCN. Combining color with luminance resulted in waveform and difference wave patterns equivalent to those of achromatic stimuli. When large levels of chromaticity contrast were added to targets with small levels of luminance contrast, PCN latency was speeded. In conclusion, the mechanisms underlying attentional salience are constrained by the low-level inputs they receive. Furthermore, speeded PCN latencies for stimuli that combine color and luminance signals compared to stimuli that contain luminance alone demonstrate that color and luminance channels are integrated during pre-attentive visual processing, before top-down allocation of attention is triggered.

## Introduction

The processing of color occurs in multiple stages. It begins with the absorption of light by three different types of retinal cone cells and continues through combining and contrasting the resulting neural signals within the retina and, subsequently, the lateral geniculate nucleus (LGN), giving rise to three retinogeniculate pathways (Dacey & Packer, 2003; Lee, 2011). The first of these combines the L and M (L+M and –L–M) signal changes, coding luminance. The second contrasts them (L–M and M–L), producing a reddish-greenish dimension. The third contrasts the S cone activity with the combined activities of the L and M cones [S–(L+M)] and [(L+M)–S], generating a bluish-yellowish dimension (Derrington, Krauskopf, & Lennie, 1984). As L+M involves combining and L–M and S–(L+M) involve contrasting the relative activity of cones, they are referred to as “cone-additive” and “cone-opponent” color mechanisms, respectively. These three mechanisms feed information into the cortex, where later stages of color processing give rise to the more conventional color representations of red-green and blue-yellow (“color-opponent processing”).

A long-standing debate in color attention research concerns the degree to which the two types of color representations direct attentional color selection. Different studies implicate one level of processing over the other. The findings of D'Zmura (1991) and Stroud, Menneer, Cave, and Donnelly (2012) suggest that attentional selection is hue (color-opponent) based, whereas the studies by Nagy and Thomas (2003) and Lindsey, Brown, Reijnen, Rich, Kuzmova, and Wolfe (2010) show cone-opponent-based attentional selection (for an overview, see Martinovic & Hardman, 2015). Retinogeniculate mechanisms (i.e., the cone-additive and cone-opponent color mechanisms) impose constraints on more familiar perceptual dimensions of color, such as saturation (Schiller, Valsecchi, & Gegenfurtner, 2017). Thus, it is not surprising that multiple color mechanisms impact attentional selection. Also, color and luminance contrast are processed interdependently, both in terms of perception and in terms of attentional selection (Martinovic & Andersen, 2018). Such co-dependence of color and luminance processing is not surprising, as there are “combination” or “conjunctive” neurons in V1 (the primary visual cortex) that are tuned to both color and luminance contrast (Shapley & Hawken, 2011; Thorell, de Valois, & Albrecht, 1984). Indeed, various types of combination neurons have been found (Nothdurft, 2000). This makes a great deal of sense evolutionarily, as features are rarely presented in isolation in natural scenes, and combination neurons would provide a shortcut to process multiple features using a single neuron. This is particularly true of color and luminance and is reflected in the much larger number of combination neurons compared to color- or luminance-only neurons (Thorell et al., 1984).

One theory of attentional salience that takes into account the presence and location of these combination neurons, as well as the intertwined nature of various visual features, is that of Zhaoping and Snowden (2006). These authors postulate that visual salience is computed in V1 through the formation of a salience map, where salience is determined for each part of the visual field and the highest point of activity determines where attention is deployed (see also Koch & Ullman, 1985). For example, the visual field corresponds to an *x*-*y* plane, and the *z*-axis represents the level of salience. Attention is allocated to the *x*-*y* position with the highest *z*-value. In terms of neuronal structure, each position on the visual field is mapped to a specific cluster of neurons. The neurons within each cluster may be tuned to specific features (e.g., luminance polarity, color, orientation; for a full list of probable feature dimensions, see Wolfe & Horowitz, 2004) or to combinations of features (e.g., color and luminance polarity). Salience within each neuronal cluster is determined using a “winner-take-all” approach, with the most active cell representing the highest level of salience for that cluster.

Following this, attention is allocated to the point on the visual field mapped to the neuronal cluster with the highest activity; however, in the case of combination (or conjunction) neurons, the presence of multiple features of equal salience (e.g., color and luminance polarity) causes higher levels of activity for combination neurons than its relevant single-feature neurons. As such, salience would be dependent on the combination of both color and luminance polarity, rather than either feature alone. However, Zhaoping and Snowden's V1-based salience map is at odds with many other models of attention that postulate that the salience map is located in areas beyond the primary sensory cortex (e.g., V4). One such model suggests that the salience of individual features is computed over the entire visual field into individual feature contrast maps, which are summed and combined with top-down influences (likely in higher visual cortex areas such as V4) into an overall salience map. Attention is then allocated to the location within the visual field with the highest *overall* salience (Itti & Koch, 2001; Melloni, Van Leeuwen, Alink, & Müller, 2012).

The aim of the present study was to explore contrast-driven, low-level constraints on attentional salience by using color contrast only, luminance contrast only, and combined color/luminance contrast stimuli using behavioral and electroencephalographic (EEG) measures. Electroencephalography enables millisecond-by-millisecond analysis of brain electrical activity and has provided a wealth of information on the attributes of attention-related cortical processes, becoming an essential complement to behavioral methods in the study of the neural bases of attention. An event-related potential (ERP) component frequently used in visual attention research is the posterior contralateral negativity (PCN), which is also often referred to as N2-posterior-contralateral (N2pc), as it is most frequently generated at the time window of the non-lateralized N2 component of the ERP. By subtracting ERPs ipsilateral to the target's location from contralateral ERPs at parieto-occipital electrodes (PO7 and PO8), the difference waveform and difference wave components such as the PCN can be calculated.

When only one negative-going difference wave component is present, it is typically associated with the PCN. When multiple difference wave components are present, however, identification of the PCN relies on its time frame (200–300 ms post-stimulus) and the fact that it is usually derived from more negative contralateral activity compared to ipsilateral activity following the typical P1–N1–P2–N2 sequence of ERP components elicited by the stimulus. Previous studies have shown that the PCN decreases in latency and increases in amplitude as the salience of a target within a visual search is increased. Thus, it is thought to “reflect the focusing of attention on a potential target item and the filtering of the surrounding distractor items” (Luck, 2012). Recently, Töllner, Zehetleitner, Gramann, and Müller (2011) confirmed the PCN's change in concert with the salience of a visual stimulus using systematic variations in a target's salience level. As they changed the salience of the target in a visual search task from high to low (through varying either target orientation away from horizontal or varying the contrast of a single color), the latency of the PCN increased and the amplitude decreased (see also Töllner, Conci, & Müller, 2015, for similar PCN effects for shape-defined targets). This finding suggests that the PCN is likely to reflect target salience in visual pop-out searches with physically balanced, bilateral displays.

Although Töllner, Zehetleitner, Gramann et al. (2011) used both orientation and color, their stimuli consisted of colored bars presented against a black background. Colors were isoluminant to each other but not to the background. Thus, color salience was not studied in isolation, and, although a definite effect of salience level was found, differences between the various achromatic and chromatic mechanisms that underlie human vision could not be explored. A study by Pomerleau, Fortier-Gauthier, Corriveau, Dell'Acqua, and Jolicœur (2014) also used electroencephalography to measure differences in attention, although their research was focused on the differences among various hues (red, green, blue, and yellow). They found that, when presented with gray distractors, red or blue targets produced faster latencies and larger amplitudes of the PCN than did green or yellow targets, suggesting that there may be differences in attentional deployment among colors. However, the colors were not systematically selected from a certain color space and differed in terms of saturation and other properties. Again, they were isoluminant with each other but not with the background. Thus, the way in which low-level chromatic mechanisms drive attentional effects at the level of the cortex remains unclear.

Because the findings of Töllner, Zehetleitner, Gramann et al. (2011) and Pomerleau et al. (2014) did not fully separate or control for the various cone-opponent and cone-additive mechanisms that contribute to color processing, in isolation or in combination with each other, we addressed this gap in the literature in a multi-experiment study that combined color psychophysics and EEG methods. In the first experiment, we presented participants with stimuli containing L+M, L–M, or S–(L+M) signals adjusted to equal perceived salience and recorded their EEG responses to determine whether ERPs associated with attentional selection systematically change as a function of these different mechanisms. In the second experiment, we determined the effect of combining color and luminance signals by presenting stimuli containing L+M and L–M signals in isolation or combination and recording participants’ EEG responses.

It is possible to derive several predictions from existing models of color vision and visual attention. If the difference waves derived from ERPs reflect contributions from the cone-additive and cone-opponent mechanisms, then they may be different from each other. Luminance stimuli (L+M) produce shorter latencies and amplitudes than either the L–M or S–(L+M) stimuli, as seen in previous studies on latencies and amplitudes of visual-evoked potentials elicited by different retinogeniculate mechanisms (e.g., Rabin, Switkes, Crognale, Schneck, & Adams, 1994). Asymmetries are sometimes observed in the S–(L+M) mechanism, as increments (bluish) and decrements (yellowish) are processed differently from the retina onward (e.g., Tailby, Szmajda, Buzás, Lee, & Martin, 2008), and yellowish generally has increased salience and more efficient attentional selection than bluish when presented at equal contrasts (Martinovic & Andersen, 2018; Switkes, 2008; Wool, Komban, Kremkow, Jansen, Alonso, Li, & Zaidi, 2015). When it comes to the effect of combining color and luminance information on ERPs, predictions for these combination conditions differ significantly between the two aforementioned models of salience. Itti and Koch's model would predict that reaction times and ERPs should be determined by some sort of summation between luminance and color, as these two would be treated as separate feature dimensions (Wolfe & Horowitz, 2004). On the other hand, in Zhaoping and Snowden's “winner-take-all” model, reaction times and ERP activity should be determined by the more salient feature (luminance or chromaticity contrast) or should even show supra-additive effects for two features due to the activation of color/luminance combination neurons.

## Experiment 1. Neural correlates of attentional salience for cone-opponent and cone-additive signals

### Materials and methods

#### Participants

Nineteen participants (15 females; age range, 20–56 years; two participants older than 30) took part in this experiment. Three participants were removed from further analysis due to excessive noise (due to signal-to-noise ratio analysis; see the EEG recording and signal processing section) or technical issues with the EEG recording, leaving a final sample of 16 participants. All participants had normal or corrected-to-normal vision and no history of neurological or psychiatric disorders. The Cambridge Color Test was used to check for any color vision deficiencies (Regan, Reffin, & Mollon, 1994). Participants gave their informed written consent and were reimbursed for their time and effort. The experiment was approved by the University of Aberdeen's Psychology Ethics Committee and was in accordance with the Declaration of Helsinki.

#### Stimuli

Stimuli were defined in DKL color space (Derrington et al., 1984). The axes of the *x*-*y* plane (angles of rotation of 0°, 90°, 180°, and 270°) correspond to the L–M and S–(L+M) cone opponent mechanisms, with 0° and 180° referring to the ΔL–M increment (reddish) and decrement (greenish) and 90° and 270° referring to the ΔS–(L+M) increment (bluish) and decrement (yellowish), respectively. The axis on the *z* plane corresponds to luminance (L+M). At an angle of elevation of 0°, no luminance component is nominally present; the resulting *x*-*y* plane is referred to as the isoluminant plane, as all colors selected from this plane have equal amounts of luminance.

**Figure 1.**
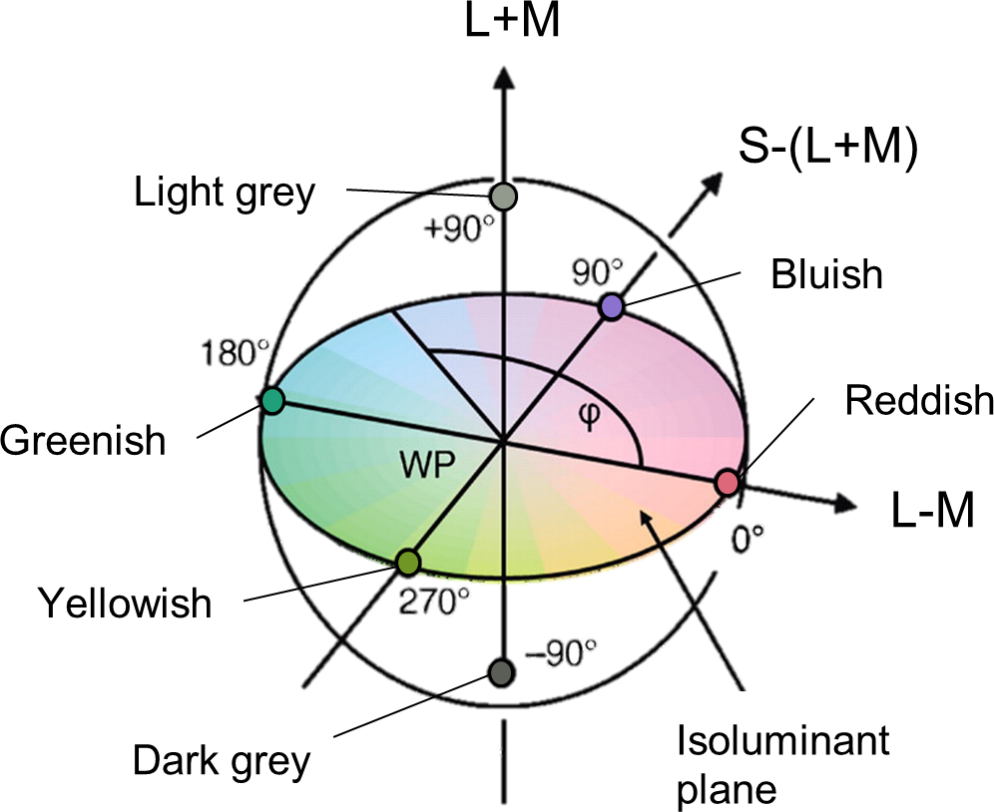
DKL color space and locations of the colors selected for the study: along the L–M and S–(L+M) axes (reddish, greenish, bluish, and yellowish) and along the negative (dark gray) and positive (light gray; standard) polarities of the L+M luminance axis. WP is the white point, which lies at the intersection of the three axes and is used as the background. Adapted from Figure 1 in Martinovic, Mordal, and Wuerger (2011).

As the experiment aimed to identify systematic differences in the ERP waves caused by chromatic and luminance signals (and between different chromatic signals), the stimuli used were selected from the cardinal axes of DKL color space (see Figure 1). All stimuli were presented on a neutral background (white point in Figure 1, with CIE 1931 *xyY* coordinates of 0.3002, 0.3159, and 46.38 cd/m^2^). They isolated either cone-opponent or cone-additive (L+M) mechanisms, corresponding to the reddish-greenish (L–M), bluish-yellowish (S–[L+M]), and luminance (L+M) axes. The six stimuli will be referred to as reddish and greenish, bluish and yellowish, and light and dark gray for the increment and decrement of ΔL–M, ΔS–(L+M), and ΔL+M, respectively.

**Figure 2.**
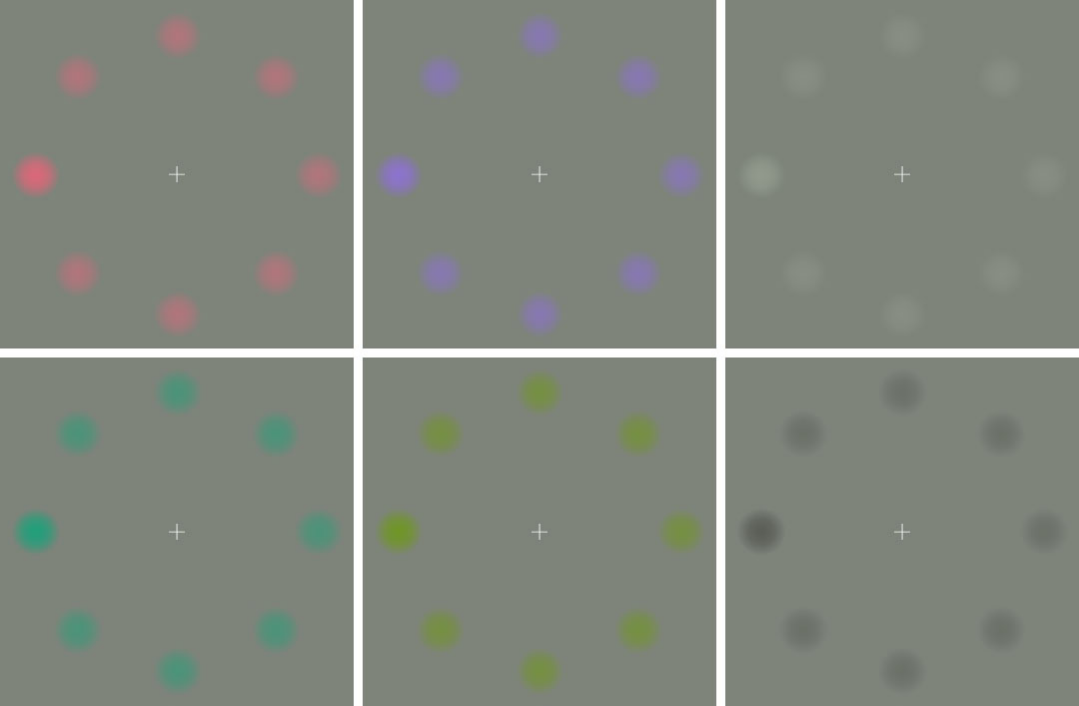
Stimulus arrays for the search task. The columns depict reddish/greenish, bluish/yellowish, and light gray/dark gray. In each array, the Gaussian position on the left side of the horizontal axes is higher in contrast. This Gaussian is the target, with all other Gaussians representing distractors. For the light gray array, they were set to a fixed contrast ratio of 2:1. For all the other arrays, contrasts were set to a level achieved in a preliminary perceived contrast adjustment task, with distractors being adjusted to be isosalient with the light gray distractors and targets being adjusted to be isosalient with the light gray target (see the Baseline experiment section for details).

The stimuli used in the experiment consisted of eight Gaussians (size = 1.2° visual angle; Gaussian deviation = 20% of stimulus size) arranged in a centered, circular array 3° visual angle from the center of the screen against a neutral background (Figure 2) displayed on a ViewSonic P227f monitor (Brea, CA). The circles were arranged such that there were Gaussians on the horizontal and vertical axes, with the remaining four Gaussians at 45° in between the four horizontal and vertical Gaussians.

#### Luminance adjustment: heterochromatic flicker photometry

To remove luminance as a confound from the chromatic stimuli, reddish, greenish, bluish, and yellowish were first adjusted to isoluminance for each participant individually using heterochromatic flicker photometry (Walsh, 1958). The stimulus display flickered between either reddish and greenish or bluish and yellowish at a rate of 20 Hz. A Cedrus RB-530 Button Box (San Pedro, CA) was used to alter the luminance of the colors. Participants were instructed to adjust the luminance of the stimuli until a minimum amount of flicker was perceived. At this point, the two colors were isoluminant. The step sizes were ±0.008 Weber luminance contrast for the bluish/yellowish condition and ±0.014 Weber luminance contrast for the reddish/greenish condition. The degree of elevation displayed at the beginning of each trial for each condition was randomized (trial-by-trial) within the range of 0 ± 0.041 for the bluish/yellowish condition and ±0.070 for the reddish/greenish condition. The level of individual variation is much higher for the L–M mechanism, thus the higher range. Eight trials were recorded per condition, with participants completing all trials for the bluish/yellowish condition followed by all trials for the reddish/greenish condition. The trials with the highest and lowest values were discarded, and the averages from the remaining six trials were used to adjust experimental stimuli to isoluminance.

#### Baseline experiment: behavioral salience adjustment

Previous research has documented differences in salience, both among participants and among different color directions within the same participant (Switkes, 2008; Switkes & Crognale, 1999). In our experiment, the aim was to compare neural and behavioral correlates of salience for stimuli belonging to different mechanisms. Therefore, to remove baseline salience differences as a confounding factor, participants first adjusted the salience of the chromatic stimuli and dark gray to equal perceived salience with light gray at low levels of contrast (Lum_L_; Weber contrast, 0.095; CIE 1931: 0.3023, 0.3201, and 51.01cd/m^2^) and high levels of contrast (Lum_H_; Weber contrast, 0.19; CIE 1931: 0.3025, 0.3203, and 55.48 cd/m^2^).

The stimulus display consisted of eight Gaussians, as described above in the Stimuli section. In the baseline experiment, seven of the Gaussians were fixed to either Lum_L_ or Lum_H_. The eighth Gaussian was the target of the adjustment procedure and could be reddish, greenish, bluish, yellowish, or dark gray, appearing randomly at one of the lateral positions (not the top or the bottom) around the circular display. A Cedrus RB-530 Button Box (San Pedro, CA) was used to increase (left) or decrease (right) the contrast of the target. Participants were instructed to match the perceived contrast of the target to the perceived contrast of the seven light gray Gaussians to achieve equal salience.

The approximate step sizes for each condition were ±0.0017 mechanism contrast for reddish and greenish, ±0.020 mechanism contrast for bluish and yellowish, and ±0.012 for dark gray. These step sizes were chosen based on detection thresholds and just noticeable differences for stimuli from the reddish/greenish and bluish/yellowish mechanisms (Gagin et al., 2014; Jennings & Barbur, 2010). It was intended that the initial contrast of the target stimulus would be randomized as (initial contrast ± 5) × step size, but an error in coding resulted in a fixed initial contrast being used for all trials. Thus, initial contrasts displayed at the beginning of each trial were 0.017 for reddish and greenish, 0.15 for bluish and yellowish, and 0.037 for dark gray. This lack of randomization was not noticed by the experimenters when piloting the task, and participants were (mistakenly) informed that the initial contrast would be randomized. Therefore, it is unlikely participants used memorization of previous responses to perform subsequent trials in the task. Participants matched reddish, greenish, bluish, yellowish, and dark gray in salience to lower and higher contrast light gray, with each condition repeated ten times. The order of conditions was randomized for each participant. The highest and lowest matches were excluded for each stimulus. The mean contrast calculated from the remaining eight trials was used to set up the experimental stimuli for the EEG experiment. The data from this salience adjustment task may be found in the Supplementary Material.

#### Main experiment: EEG

The EEG experiment used the same stimulus array as the salience adjustment task. One of the Gaussians (the target) had the contrast of the higher contrast stimulus from the adjustment experiment, and the other seven Gaussians were homogeneous distractors of the same color mechanism and direction as the target (reddish, greenish, bluish, yellowish, and light or dark gray), which were fixed at the lower contrast from the adjustment experiment. For light gray, these were fixed contrast values to which the matches were performed and were thus the same across participants; for the other five conditions, these were individually adjusted contrast values. See Figure 2 for examples of the six conditions.

**Figure 3.**
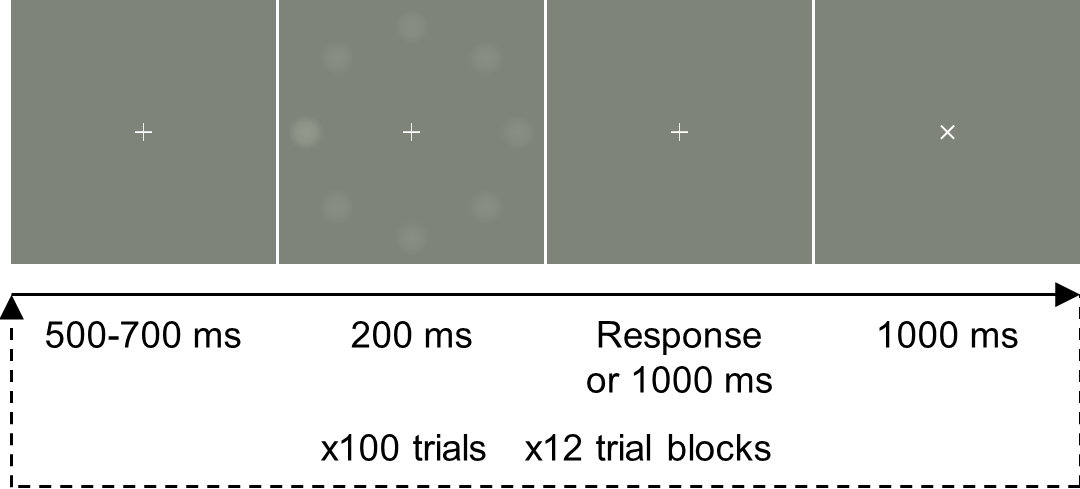
Trial sequence, showing the timing of a single trial; 1200 trials were recorded in 12 blocks.

A flowchart of the visual search task is shown in Figure 3. A trial consisted of 500 to 700 ms of a fixation cross, followed by a 200-ms presentation of the stimulus. The participants were then given a maximum of 1000 ms to respond, indicating (via the left or right button) on which side of the visual field the target was located. Following the response, a 1000-ms intertrial interval was given (shown as an X on the screen). The next trial (starting with presentation of the fixation cross) followed immediately. The experiment was divided into 12 blocks, each containing 100 trials. The order of trials was randomized for each participant, with a total of 200 trials (100 right and 100 left targets) recorded for each of the six conditions.

At least one practice block of 20 trials was administered before the start of the recording to familiarize participants with the task. The practice block was repeated until the participant scored greater than 75% accuracy for two subsequent practice blocks, which generally required two blocks only. Feedback on accuracy was given after each block.

#### EEG recording and signal processing

Electroencephalographs were continuously recorded from 64 scalp sites at a sample rate of 256 Hz using a BioSemi ActiveTwo system (Amsterdam, Netherlands). Electrodes were mounted on a BioSemi headcap, with positions corresponding to the 10-20 electrode system (Jasper, 1958). External electrodes were placed at the outer canthi of the eyes and the superior and inferior orbits of the left eye to measure eye movements and blinks. Data were epoched (200 ms pre-stimulus to 500 ms post-stimulus) and low-pass filtered at 40 Hz. Baseline activity was taken from 200 to 0 ms pre-stimulus.

The automated procedures FASTER and ADJUST were used for artifact rejection, with the data referenced to the Fz electrode for FASTER and an average reference for ADJUST. FASTER removed global channel and epoch artifacts based on the *Z* scores of various parameters such as the mean correlation coefficient between channels, the amplitude range within an epoch, and the variance within channels or epochs. Independent component analysis (ICA) was carried out after the rejection of globally contaminated trials using ADJUST. The program decomposes the EEG data into independent components (ICs) and generates spatial and temporal criteria for each of four artifact categories (blinks, discontinuities, and horizontal and vertical eye movements). Each IC is analyzed based on these criteria. If an IC exceeds the thresholds set for all criteria from one of the artifact categories (e.g., blinks), the algorithm classifies that IC as that artifact (blink) and removes the component from subsequent analysis. FASTER was then used to interpolate global and local channel artifacts (e.g., transient electrical drifts), again using the *Z* scores of various parameters (e.g., amplitude range, variance, median slope). For a more in-depth explanation of the FASTER and ADJUST processes, see Nolan, Whelan, and Reilly (2010) and Mognon, Jovicich, Bruzzone, and Buiatti (2011), respectively. The average overall trial rejection rate was 4.89% (59 trials). The average total ICA components rejected were 12 per participant, with varying rates for the four IC categories: blinks (average < 1), discontinuities (average = 7), horizontal eye movements (average = 1), and vertical eye movements (average = 3). Finally, incorrect trials and trials with artifacts and (potentially remaining) blink- and eye-movement-associated activity (criterion: 25-µV deviation in vertical or horizontal electrooculogram) were excluded from subsequent analyses.

Successful artifact rejection was confirmed with subsequent visual inspection. Data were average referenced after artifact rejection. Finally, signal-to-noise ratio (SNR) analysis was used to identify and remove those conditions in which the ERP signal was not reliable within the time windows of interest. To identify unreliable data, the global field power was calculated for each recorded time point and assessed if it was significantly different from random noise, which was obtained by randomizing the data (shuffling the data among the 64 EEG channels) and recalculating the global field power values. The threshold *p* value for a false discovery rate of 5% was calculated, and conditions that had *p* values equal to or exceeding the threshold *p* value within the time window of interest were removed from analysis. For an in-depth explanation of this procedure, see Koenig and Melie-García (2010). This SNR procedure is a useful approach in situations when relatively low-contrast stimuli lead to low amplitude ERPs (see Jennings & Martinovic, 2015), as it allows the removal of such noisy data points before statistical testing.

To identify the PCN wave (and any other event-related EEG lateralization) that may be present, difference waves were calculated by subtracting ERPs ipsilateral to the target from contralateral ERPs at channels PO7/8. Amplitudes and peak latencies of the various difference wave components were calculated using the same methods as in Töllner, Zehetleitner, Gramann, et al. (2011); latencies were determined individually as the time points of maximum deflection (positive or negative) within the time windows 100 ms to 300 ms post-stimulus, respectively. ERP amplitudes were calculated by averaging the 20-ms window around the maximum deflection.

#### Statistical data analysis

The reaction times (RTs) and accuracies were compared using two 3 × 2 repeated-measure ANOVAs with factors of mechanism (reddish/greenish, bluish/yellowish, and light gray/dark gray) and color direction (increment: reddish, bluish, and light gray; decrement: greenish, yellowish, and dark gray). Significant effects were further examined using Bonferroni-corrected post hoc comparisons and paired-sample *t*-tests. When Mauchly's test of sphericity showed significant violations, the results of ANOVAs were reported using Greenhouse–Geisser corrections. The latencies and amplitudes of the difference wave components were subjected to the same statistical analyses as the behavioral data.

### Results

#### Behavioral results: RTs and accuracies

**Figure 4.**
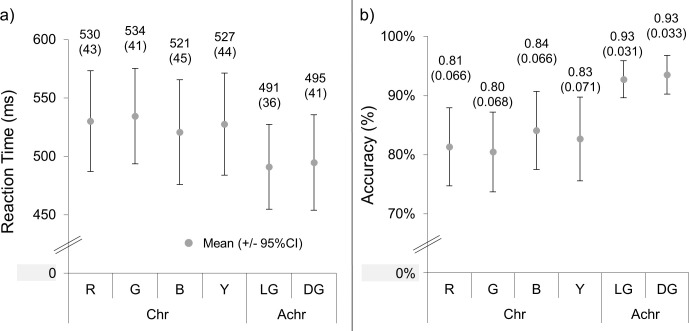
The RTs (a) and accuracies (b) of the search task in Experiment 1. R, G, B, Y, LG, and DG correspond to the reddish, greenish, bluish, and yellowish (chromatic, Chr) and light gray and dark gray (achromatic, Achr) conditions, respectively. The means and 2 standard errors (in brackets) are above each data point. The units are milliseconds and percent for the RTs and accuracies, respectively.

The mean RTs and accuracies and their 95% confidence intervals are shown in Figure 4. Two 3 × 2 repeated measures ANOVAs (with factors of mechanism and color direction) were performed for the RT and accuracy results. A significant effect of mechanism was found for both RTs (*F*(2,30) = 12.6, *p* < 0.001, η*_p_*^2^ = 0.46) and accuracies (*F*(2,30) = 10.8, *p* < 0.001, η_*p*_^2^ = 0.42), with post hoc comparisons (Bonferroni-corrected *p* = 0*.*017) showing that light gray/dark gray had faster RTs (*t*(15) = 4.53; *p* < 0.001) and higher accuracies (*t*(15) = 4.67; *p* < 0.001) than reddish/greenish (RT: *t*(15) = 4.53, *p* < 0.001; accuracy: *t*(15) = 4.67, *p* < 0.001) and bluish/yellowish (RT: *t*(15) = 4.16, *p* = 0*.*001; accuracy: *t*(15) = 3.17, *p* = 0*.*006). Reddish/greenish and bluish/yellowish were not significantly different from each other for RTs (*t*(15) = 0.96; *p* = 0*.*35) and accuracies (*t*(15) = –1.26; *p* = 0*.*23). There was no significant effect of color direction for the RTs (*F*(1,15) = 1.07; *p* = 0*.*32, η*_p_*^2^ = 0.066) or the accuracies (*F*(1,15) = 0.090; *p* = 0*.*77; η*_p_*^2^ = 0.006), nor was there an interaction between the two factors (RT: *F*(1.41,21.1) = 0.052, *p* = 0*.*95, η*_p_*^2^ = 0.003; accuracy: *F*(2,30) = 0.41, *p* = 0*.*67, η*_p_*^2^ = 0.027).

#### ERP analysis

**Figure 5.**
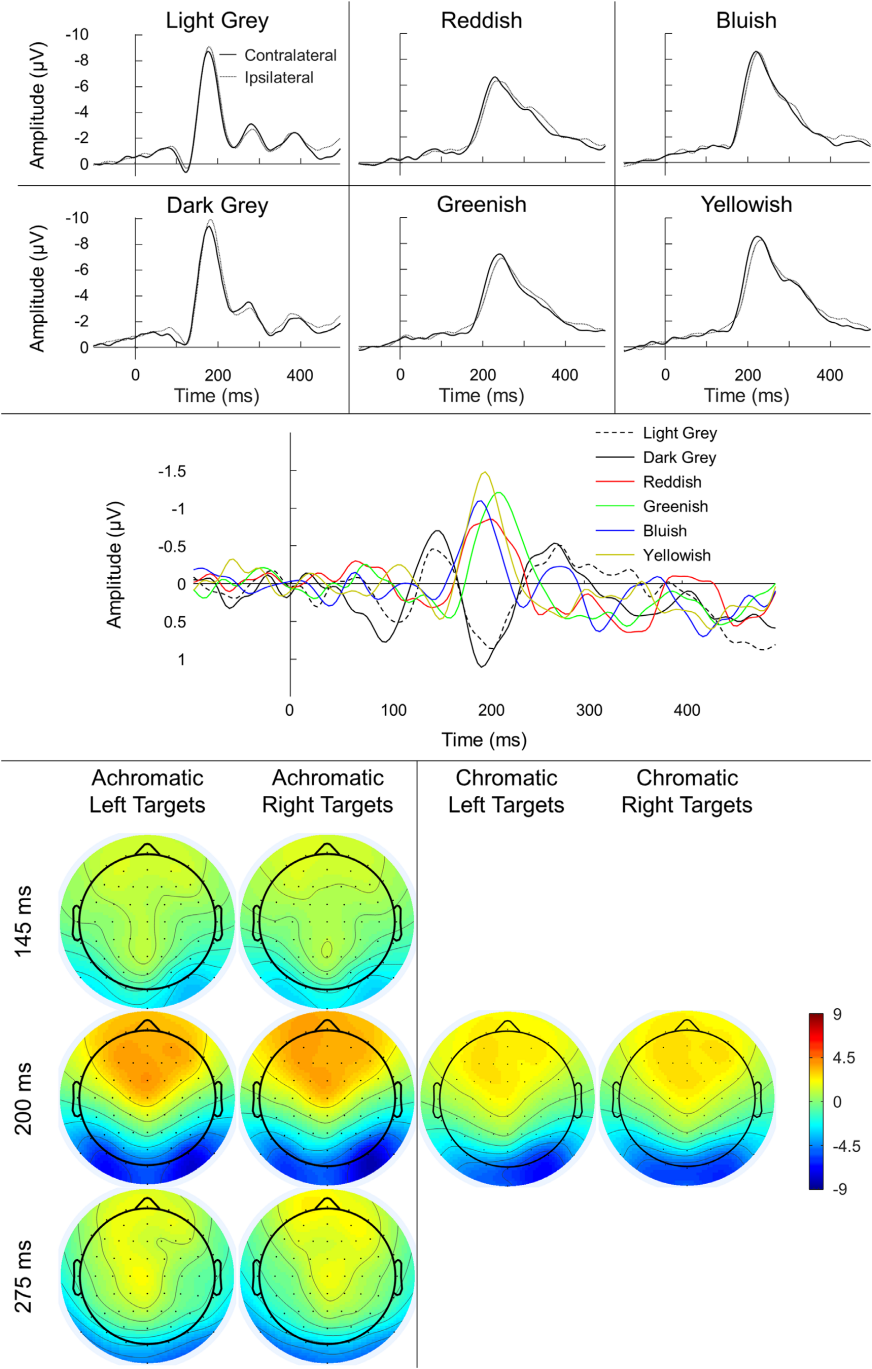
Grand-average contra- and ipsilateral waveforms (to the target location) at PO7/8 channels (top), grand-mean difference waveforms (calculated by subtracting ipsilateral from contralateral activity; middle) and topographical maps of ERPs (bottom) for the six conditions of Experiment 1. Negativity is plotted upward for the contralateral/ipsilateral and difference waveform graphs. Note the presence of three difference wave components for the achromatic conditions compared to the single component present for the chromatic conditions. The topographical maps represent the activity (in µV) across the entire scalp at latencies approximately equal to the peaks of three (achromatic conditions) and single (chromatic conditions) difference wave components.

The reddish data from two participants, greenish data for one participant, and yellowish data for one participant were excluded from this analysis due to inadequate SNR. The grand-mean contra- and ipsilateral waveforms and the difference waveforms are shown in Figure 5. The luminance conditions produced strong, steep negative deflections in both contra- and ipsilateral PO7/8 electrodes. Likewise, the chromatic conditions also produced negative deflections in both contra- and ipsilateral electrodes, although the deflections occurred later than for the luminance. In the difference waves, the outcome is such that for luminance-defined targets, interestingly, we observe a sequence of difference wave components. The first of these components occurs early (∼145 ms) and manifests as a negative deflection in the difference wave; thus, we labeled it an early PCN. Next, there is a positive deflection (∼200 ms), which we labeled as posterior contralateral positivity (PCP), although we note that the time window is somewhat later to previously observed contralateral positivities (Corriveau et al., 2012; Pomerleau et al., 2014). The final component corresponds to a late PCN (∼275 ms). For the isoluminant targets, we observed a single component, resultant from an enhanced negativity contralaterally to the target (∼200 ms). Although there are distinct differences between luminance-defined and isoluminant ERPs and difference waves, EEG activity appears largely similar within the two groups themselves.

**Figure 6.**
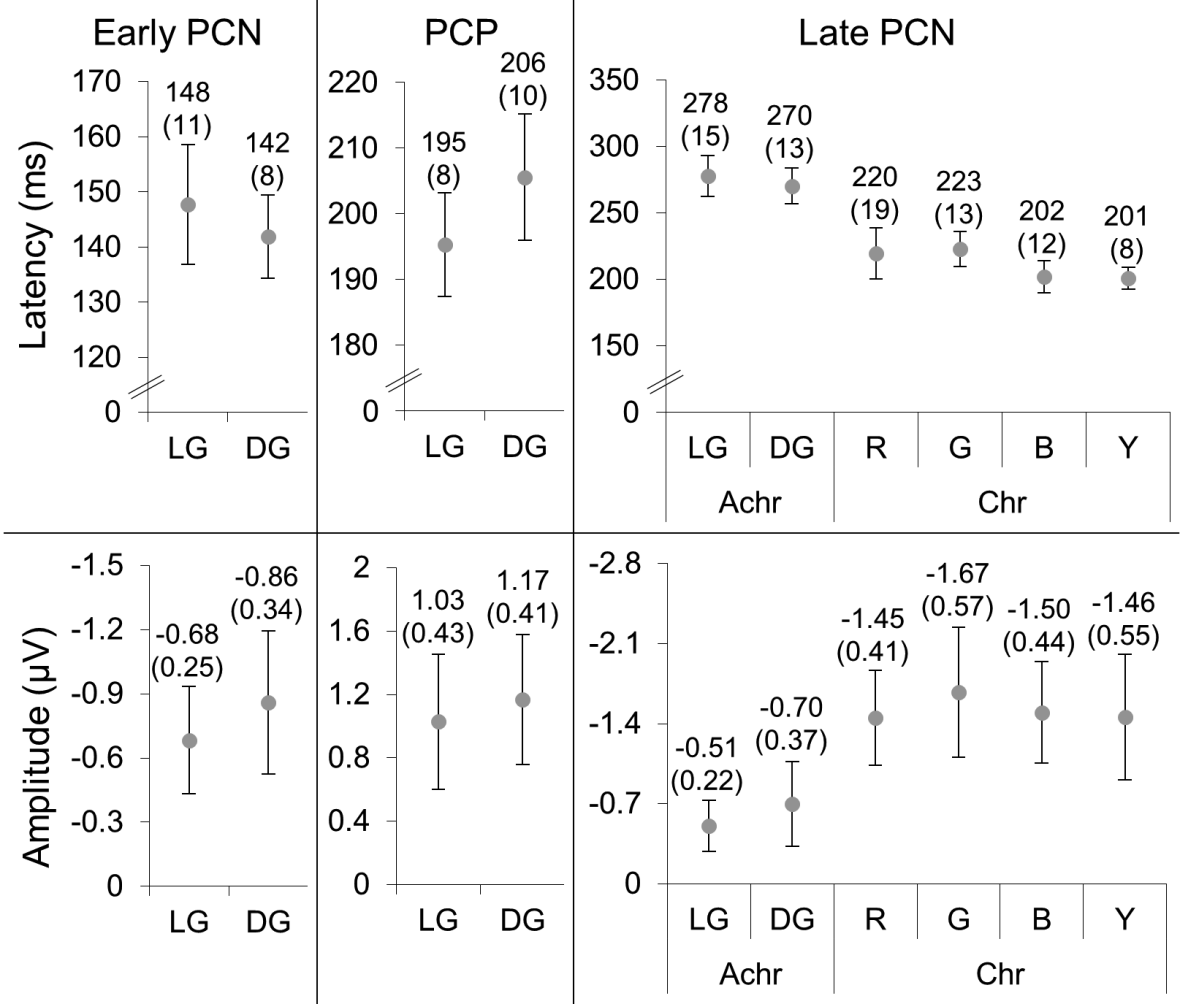
The latencies (top) and amplitudes (bottom) of the early PCN, PCP, and late PCN (left to right) detected in Experiment 1. The means and 2 standard errors (in brackets) are above each data point. The units are milliseconds and microvolts for the latencies and amplitudes, respectively.

The mean peak latencies and amplitudes of the difference wave components in the PCN and PCP windows and their 95% confidence intervals are shown in Figure 6. Paired *t*-tests between light gray and dark gray showed there were no differences between peak latencies and between amplitudes for either the early PCN (latencies: *t*(15) = 1.13, *p* = 0*.*28; amplitudes: *t*(15) = 1.00, *p* = 0*.*33) or PCP (latencies: *t*(15) = –2.08, *p* = 0*.*056; amplitudes: *t*(15) = –0.62, *p* = 0*.*54) components.

For the late PCN components, a 3 × 2 repeated-measures analysis of variance (ANOVA) showed that there was a significant effect of mechanism for peak latencies (*F*(2,22) = 27.1; *p* < 0.001, η*_p_*^2^ = 0.71) and amplitudes (*F*(2,22) = 11.7; *p* < 0.001; η*_p_*^2^ = 0.52). Post hoc analysis (Bonferroni-corrected *p* = 0*.*017) of latencies showed light gray/dark gray stimuli elicited significantly slower PCNs than either reddish/greenish (*t*(12) = 4.07; *p* = 0*.*002) or bluish/yellowish (*t*(14) = 8.67; *p* < 0.001) stimuli, which had comparable PCN latencies (*t*(11) = 2.73; *p* = 0*.*020). Post hoc analysis of amplitudes showed that light gray/dark gray PCN amplitudes were significantly less negative than either reddish/greenish (*t*(12) = 5.11; *p* < 0.001) or bluish/yellowish (*t*(14) = 3.42; *p* = 0*.*004), which were comparable (*t*(11) = –0.52; *p* = 0*.*62). No significant effect of color direction was found for either latencies (*F*(1,11) = 0.060; *p* = 0*.*81; η*_p_*^2^ = 0.005) or amplitudes (*F*(1,11) = 1.12; *p* = 0*.*31; η*_p_*^2^ = 0.092), nor were there interactions between color mechanism and color direction (latencies: *F*(2,22) = 0.16, *p* = 0*.*85, η*_p_*^2^ = 0.014; amplitudes: *F*(2,22) = 0.49, *p* = 0*.*62, η*_p_*^2^ = 0.043).

Although the lack of differences between the chromatic PCN amplitudes may seem surprising based on visual inspection of the ERP difference waves (Figure 5, middle panel), this was due to the larger amount of individual variability, particularly for the reddish condition compared to the yellowish condition (see Supplementary Material for graphs of both individual participants’ ERPs and the grand-mean ERP for each of the six conditions).

### Discussion

Reddish, greenish, bluish, yellowish, and dark gray were matched for perceived contrast with light gray to create two sets of isosalient stimuli: a low and a high contrast set. These matches were then used to generate visual search displays for an EEG experiment to determine the neural markers of attentional selection driven by cone-opponent color mechanisms as opposed to the cone-additive luminance mechanism. The task required searching for a higher contrast target among lower contrast distractors.

The difference waves for color and luminance targets appear to contain unique patterns of components. For achromatic targets, the first negativity in the difference wave occurs in a relatively early time window. It is followed by a positive deflection and a second negative dip. As argued by Corriveau et al. (2012), unbalanced displays are essential when testing subcomponents of perceptual and attentional processing. However, they sometimes lead to the emergence of early difference wave components that reflect a spatial “attend-to-me” signal (e.g., the Ppc in Pomerleau et al., 2014). Therefore, in this type of selective attention task, the early PCN may be interpreted as reflecting an *initial allocation of perceptual resources*—that is, the activation of neurons driven by increased contrast of the target relative to the distractors. Conversely, the late PCN may reflect an additional top-down *target selection* subprocess following the bottom-up-driven early PCN. The early PCN emerges at the onset of the N1 window, and the late PCN coincides with the N2 window of the ERP. Whereas N1 is associated with visual discriminative processes, N2 is taken to reflect stimulus categorization (Luck, 2014). It is also possible, however, that this complex of two PCNs and one PCP may simply reflect the asymmetry in early sensory, non-lateralized ERPs (e.g., P1, N1, P2, N2) that are well known when using stimulus arrays of varying luminance (i.e., physical energy) across hemifields. However, we do not believe that this is likely, as our waveforms do not resemble those elicited by simple, lateralized stimulation (Johannes, Münte, Heinze, & Mangun, 1995). Also, our accuracies are not at ceiling, reflecting that the difference in contrast is relatively small; therefore, our task is not overly easy and requires non-trivial amounts of perceptual-attentional resources, whose allocation should have a significant impact on the resulting difference waves.

For chromatic displays, by contrast, there is only a single negative-going deflection reflecting the PCN. The chromatic PCN occurs at a latency that falls between the luminance-driven early and late PCNs. Both the difference and contralateral/ipsilateral waveforms of chromatic displays were significantly different from those of achromatic displays. These dissimilarities make comparing chromatic and achromatic difference waveforms difficult. This also confirms that there is a fundamental difference between the processing of color and that of luminance when they are displayed in isolation (Rabin et al., 1994). Rather than the three difference wave components present for achromatic displays, the single deflection of chromatic displays suggests a single process that enables distinction of the target from distractors.

In line with the earlier emergence of attentional EEG effects for achromatic targets, RTs and accuracies were also significantly faster and higher (respectively) for luminance-only compared to color-only conditions. The equal latencies and amplitudes of the early PCN for light gray and dark gray were also reflected in equal RTs and accuracies. The presence of equivalent early PCN components for achromatic targets, as well as the equal RTs, latencies, and amplitudes for the PCNs of the two chromatic mechanisms, is consistent with the hypothesis that color salience is defined by cortical rather than by pre-cortical (cone-opponent) processing. When the asymmetries in the S–(L+M) mechanism were accounted for by salience matching, we failed to observe any differences between bluish and yellowish. In fact, Wool et al. (2015), who observed faster RTs and earlier local field potentials to the yellowish stimuli of nominally equal chromaticity contrast with bluish stimuli, discussed the likelihood that the greater response compression in the bluish channel correlates with lower perceived saturation, which results in reduced salience. Our results are in line with this interpretation.

In conclusion, attentional salience processing of achromatic conditions appears to occur in multiple stages, reflected in the three difference wave components. In contrast, chromatic conditions (those without any luminance) appear to be dependent on a single stage. This divide is confirmed by the RTs, latencies, and amplitudes, as the RTs and patterns of ERP activity are divided into luminance and chromaticity groups.

## Experiment 2. Salience of combined chromatic and luminance signals

The second experiment aimed to determine the effect various combinations of color and luminance contrast would have on the RTs and difference waves and, therefore, the salience of the target stimulus. It is generally expected that conditions with smaller differences in contrast relative to the distractors (and thus lower salience) will have significantly slower latencies (and lower amplitudes) than the conditions with higher contrast differences (and thus higher salience). More specifically, we want to compare the difference wave components to assess how they respond to changes in target salience when driven by color, luminance, or combined color and luminance processing. Combining luminance and color in one stimulus will allow us to assess the degree to which difference waves elicited by such contrast combinations relate to difference waves driven by luminance-isolating or color-isolating stimuli. Previous ERP studies on attentional salience (e.g., Girelli & Luck, 1997; Hillyard & Münte, 1984; Vierck & Miller, 2008; Wijers, Lange, Mulder, & Mulder, 1997) all relied on stimuli that combined color and luminance, but they did not co-vary their content systematically. Thus, our study will extend insights into low-level determinants of attentional selection obtained in Experiment 1 and provide support for the theories of salience processing of either Zhaoping and Snowden (2006) or Itti and Koch (2001).

### Materials and methods

#### Participants

Eighteen participants (13 female; 16 right-handed; age range, 19–56 years; *M* = 26.5) took part in this experiment. Six had previously taken part in Experiment 1. All participants had normal or corrected-to-normal vision and no history of neurological or psychiatric disorders. As with Experiment 1, all participants were screened for color deficiencies using the Cambridge Color Test (Regan, Reffin, & Mollon, 1994). Participants gave their informed written consent and were reimbursed for participating. The experiment was approved by the University of Aberdeen's Psychology Ethics Committee and was in accordance with the Declaration of Helsinki.

#### Stimuli

The basic stimulus display was the same as in Experiment 1, with a visual search for higher contrast targets (see Figure 1). The background had CIE1931 coordinates of 0.3052, 0.3154, and 42.1 cd/m^2^. In this experiment, we used the same fixed contrast levels for all participants. Adjusting perceived contrast for color/luminance combined stimuli would pose problems, because they could be manipulated both along the luminance and along the color dimension. We selected a color and a luminance direction for use in the experiment based on conditions with the most stable results in Experiment 1; the criteria were based on a combination of the largest amplitude, least inter-participant latency variability and noise within the difference waveforms, smallest number of removed observations, and greater monitor gamut range. These were found to be light gray and greenish. Seven distractor Gaussians were homogeneous distractors, as in Experiment 1, with the contrast of either the individual mechanisms (1_L_ or 1_C_) or the combination of both (1_L_/1_C_), depending on the condition. The distractors were equivalent to the low contrast light gray and greenish, averaged over all participants, in Experiment 1. On average, this should ensure equal salience of color and luminance distractors. Two contrast ratios were then used to generate targets, which were twice and three times the contrast of the distractors; multiples higher than three could not be used as they would have put the chromatic stimulus outside the gamut of the monitor. These conditions will be referred to as color only with twice the contrast of distractors (2_C_), luminance only with twice the contrast of distractors (2_L_), color only with three times the contrast of distractors (3_C_), and luminance only with three times the contrast of distractors (3_L_). Three combination stimuli were created from these luminance and chromatic stimuli: triple contrast in luminance and double contrast in chromaticity (3_L_/2_C_), double contrast in luminance and triple contrast in chromaticity (2_L_/3_C_), and triple contrast in both luminance and chromaticity (3_L_/3_C_). The distractors for these conditions represented a combination of the luminance and chromaticity of the light gray and greenish used for the distractors in the isolating conditions, which could be labeled as 1_L_/1_C_. This enabled analysis of differences between lower and higher salience levels within the same mechanism, between different mechanisms, and between combinations of different levels of the two mechanisms.

#### Luminance adjustment: heterochromatic flicker photometry

Using the same procedure as in Experiment 1, luminance artifacts were removed from the chromatic stimuli.

#### Procedure

We followed the same protocol as in Experiment 1, with participants instructed to indicate (via left or right button) on which side of the visual field the target was located. The EEG session was divided into 12 blocks, each containing 105 trials. There were 1260 trials in total, with 180 trials (90 right and 90 left targets) recorded for each of the seven conditions. Trials from different conditions were randomly intermixed. Practice blocks of 40 trials were administered before the start of the recording to familiarize participants with the task. Participants repeated the practice block until they scored greater than 80% accuracy for two subsequent practice blocks. For most participants this took three practice blocks; one participant took five practice blocks. Feedback on accuracy was given after each block.

#### EEG recording and signal processing

The data collection, epoching, filtering, and rejection of the EEG data were carried out in the same manner as in Experiment 1. The average rejection rate was 12.0% (151 trials). The average total ICA components rejected was 12, with varying rates for the four IC categories: blinks (average = 3), discontinuities (average = 6), horizontal eye movements (average = 1), and vertical eye movements (average = 2).

#### Statistical data analysis

To compare between salience levels and chromatic and luminance conditions, 2 × 2 repeated-measures ANOVAs, with factors of contrast type (chromaticity and luminance) and salience level (higher and lower), were performed on the RTs and accuracies. Significant effects were further examined using Bonferroni-corrected post hoc comparisons and paired-sample *t*-tests. To compare the three combination conditions to the luminance conditions and each other, paired-sample *t*-tests were performed between 2_L_ and 2_L_/3_C_, 3_L_ and 3_L_/2_C_, 3_L_ and 3_L_/3_C_, 2_L_/3_C_ and 3_L_/3_C_, and 3_L_/2_C_ and 3_L_/3_C_ (Bonferroni-corrected *p* = 0*.*010). As with Experiment 1, the contra- and ipsilateral data and corresponding difference waveforms were plotted, and the peak latencies and amplitudes of the difference wave components were subjected to the same statistical analyses as the behavioral data. When Mauchly's test of sphericity was significant, the results of ANOVAs were reported using Greenhouse–Geisser corrections.

### Results

#### RT and accuracy analysis

**Figure 7.**
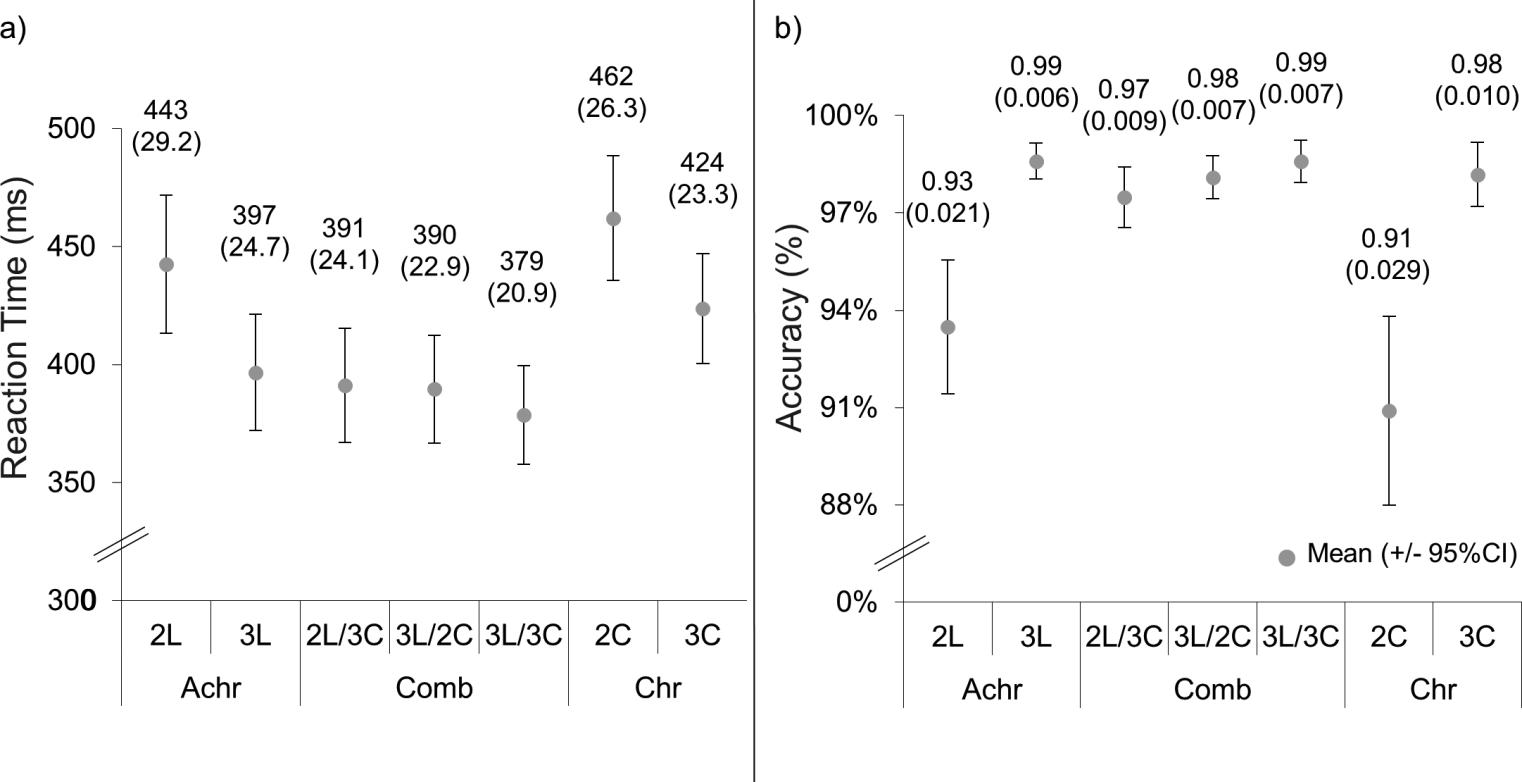
The RTs (a) and accuracies (b) of the EEG task of Experiment 2. The means and 2 standard errors (in brackets) are above each data point. The units are milliseconds and percent for the RTs and accuracies, respectively.

The mean RTs and accuracies and their 95% confidence intervals are shown in Figure 7. Accuracies and RTs from single-contrast conditions (2_L_, 3_L_, 2_C_, and 3_C_) were analyzed using a 2 × 2 repeated measures ANOVA, with the factors of contrast type (color or luminance) and salience level (lower or higher). A significant effect of contrast type was found for RTs (*F*(1,17) = 13.7; *p* = 0*.*002; η*_p_*^2^ = 0.45) but not for accuracies (*F*(1,17) = 2.87; *p* = 0*.*11; η*_p_*^2^ = 0.14). RTs for luminance conditions were faster than for the chromatic conditions. There was a significant effect of salience level for both RTs (*F*(1,17) = 142; *p* < 0.001, η*_p_*^2^ = 0.89) and accuracies (*F*(1,17) = 50.6; *p* < 0.001; η*_p_*^2^ = 0.75), with high-salience conditions having faster RTs and higher accuracies than low-salience conditions. No interaction between contrast type and salience level was found for RTs (*F*(1,17) = 1.96; *p* = 0*.*18; η*_p_*^2^ = 0.10) or accuracies (*F*(1,17) = 4.36; *p* = 0*.*052; η*_p_*^2^ = 0.20). Five paired sample *t*-tests between the 2_L_ and 2_L_/3_C_, 3_L_ and 3_L_/2_C_, 3_L_ and 3_L_/3_C_, 2_L_/3_C_ and 3_L_/3_C_, and 3_L_/2_C_ and 3_L_/3_C_ RTs showed that the combination conditions had significantly faster RTs than their equivalent luminance conditions: 2_L_ and 2_L_/3_C_ (*t*(17) = 7.75; *p* < 0.001), 3_L_ and 3_L_/2_C_ (*t*(17) = 3.17; *p* = 0*.*006), and 3_L_ and 3_L_/3_C_ (*t*(17) = 6.80; *p* < 0.001). Likewise, the 3_L_/3_C_ condition was significantly faster than both the 2_L_/3_C_ (*t*(17) = 4.84, *p* < 0.001) and 3_L_/2_C_ (*t*(17) = 5.74; *p* < 0.001) conditions. The accuracies were also significantly higher for 2_L_/3_C_ compared to 2_L_ (*t*(17) = 4.35; *p* < 0.001). There were no significant differences between 3_L_ and 3_L_/2_C_ (*t*(17) = 1.97; *p* = 0*.*066), 3_L_ and 3_L_/3_C_ (*t*(17) = 0.019; *p* = 0*.*99), 2_L_/3_C_ and 3_L_/3_C_ (*t*(17) = –2.64; *p* = 0*.*017), or 3_L_/2_C_ and 3_L_/3_C_ (*t*(17) = –1.64; *p* = 0*.*12). The lack of significant differences among the 3_L_, 2_L_/3_C_, 3_L_/2_C_, and 3_L_/3_C_ accuracies is likely due to a ceiling effect, as the accuracies of these conditions are all above 97%.

#### ERP analysis

**Figure 8.**
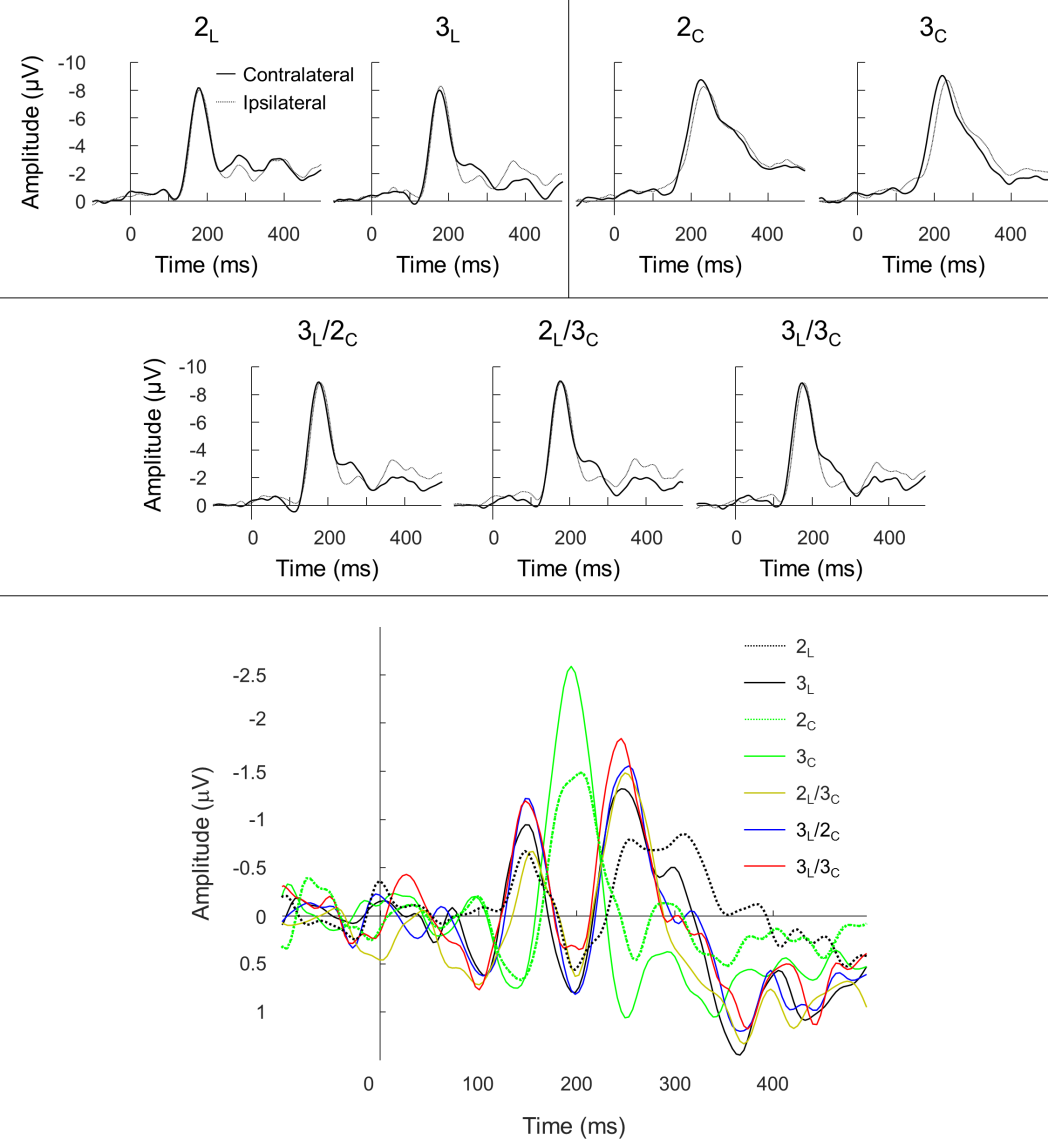
Grand-average contralateral and ipsilateral waveforms (to the target location) at the PO7/8 electrodes (top) and grand-average difference waveforms (bottom) for the seven conditions of Experiment 2. Note that negativity is plotted upwards. For the contralateral/ipsilateral graphs, the single-feature conditions are on the top and the three combination conditions are on the bottom. Note the faster latencies and steeper slopes of the posterior negativity in luminance and combination conditions compared to chromatic conditions. For the difference waveforms, note the distinct difference waveform pattern for luminance and combination conditions compared to the chromatic conditions.

**Figure 9.**
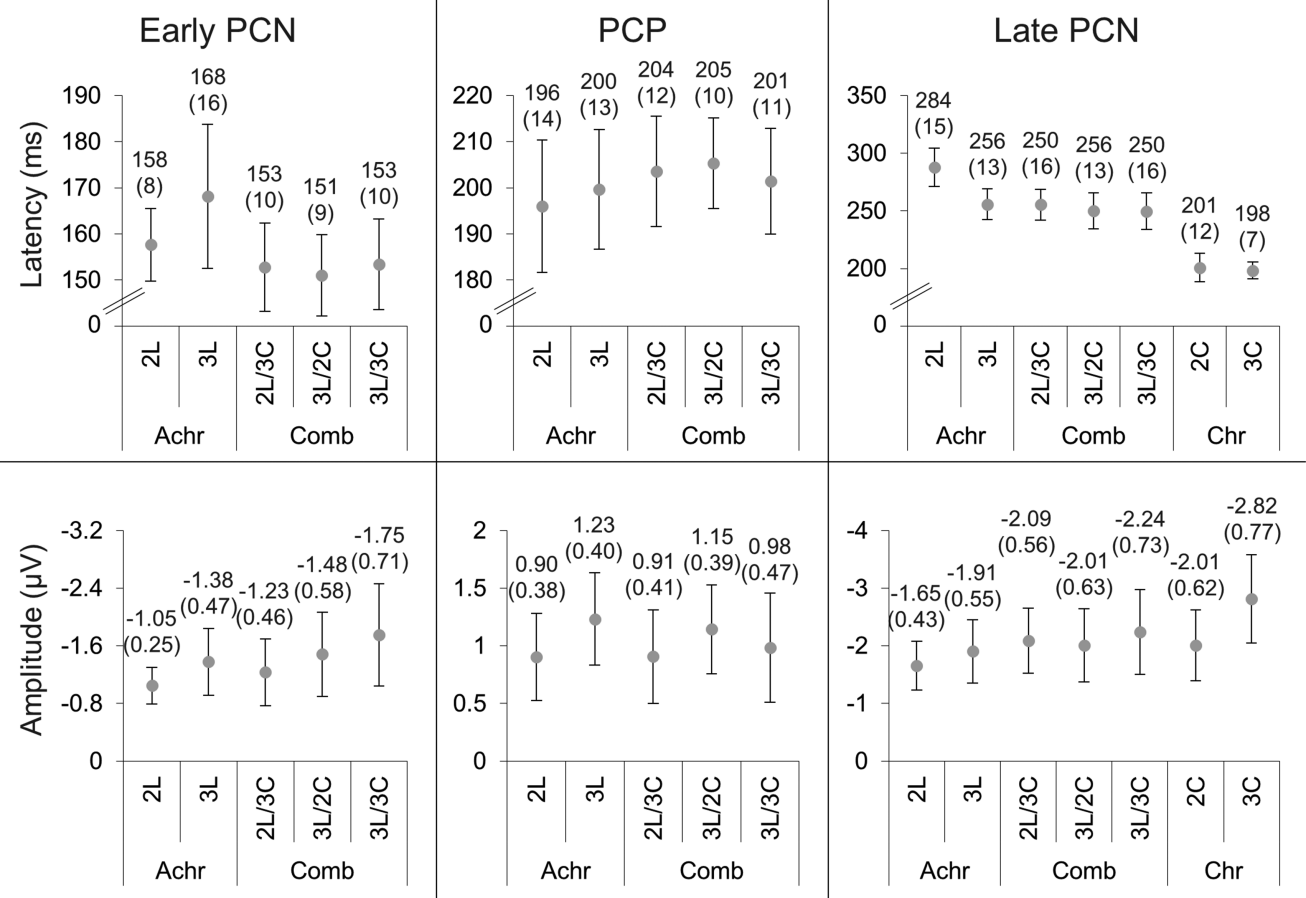
The latencies (top) and amplitudes (bottom) of the early PCN, PCP, and late PCN (left to right) detected in Experiment 2. The means and 2 standard errors (in brackets) are above each data point. The units are milliseconds and microvolts for the latencies and amplitudes, respectively.

The 2_L_ early PCN for one participant was rejected based on SNR analysis. The contra- and ipsilateral waveforms and the difference waveforms are provided in Figure 8, which shows activity similar to that seen in Experiment 1. Difference waves for color-isolating conditions showed only a single mid-latency PCN wave, whereas the luminance and combination conditions showed activity similar to that for the achromatic conditions, following an early PCN–PCP–late PCN sequence, indicating that the differences in contra-/ipsilateral activity and difference waves seen between chromatic and achromatic stimuli disappear when luminance signals are combined with chromatic signals. The mean peak latencies and amplitudes of the difference wave components and their 95% confidence intervals are shown in Figure 9.

First, we analyzed latencies and amplitudes of the early PCN and PCP components. These components were only present for conditions that contained luminance signals. Paired *t*-tests between 2_L_ and 3_L_ revealed no differences for either the latencies or amplitudes of the early PCN (latencies: *t*(16) = –1.38, *p* = 0*.*19; amplitudes: *t*(16) = 1.79, *p* = 0*.*092) and PCP (latencies: *t*(17) = –0.44, *p* = 0*.*67; amplitudes: *t*(17) = –1.18, *p* = 0*.*25). Comparisons among the three combination conditions and among the combination conditions and their equivalent luminance conditions were performed. There were no significant differences for any of the five comparisons of latencies for either the early PCN (all *t* < 1.96; all *p* > 0.066) or PCP (all *t* < 1.15; all *p* > 0.27) components. There were also no significant differences in amplitude (early PCN: all *t* < 2.45, all *p* > 0.025; PCP: all *t* < 1.00, all *p* > 0.33).

A repeated measures 2 × 2 ANOVA with factors of contrast type and salience level was performed on the PCN peak latencies and amplitudes of single-contrast conditions (2_L_, 3_L_, 2_C_, and 3_C_). For latencies, a significant effect was found for contrast type (*F*(1,17) = 62.3; *p* < 0.001; η*_p_*^2^ = 0.79) and salience level (*F*(1,17) = 8.26; *p* = 0*.*011; η*_p_*^2^ = 0.33), with color-only and higher salience conditions producing faster PCN latencies than luminance-only and lower salience conditions. There was also a significant interaction between contrast type and salience level (*F*(1,17) = 8.49; *p* = 0*.*010; η*_p_*^2^ = 0.33). Paired *t*-tests between 2_L_ and 3_L_ and between 2_C_ and 3_C_ (Bonferroni-corrected *p* = 0*.*025) showed that, although 2_L_ had a significantly slower PCN latency than 3_L_ (*t*(17) = 3.61; *p* = 0*.*002), the PCN latencies of 2_C_ and 3_C_ were equivalent (*t*(17) = 0.43; *p* = 0*.*67). Amplitudes showed a significant effect of contrast type (*F*(1,17) = 6.41; *p* = 0*.*022; η*_p_*^2^ = 0.27) and salience level (*F*(1,17) = 10.8; *p* = 0*.*004; η*_p_*^2^ = 0.39), with color-only and higher salience conditions having significantly larger amplitudes than the lower salience conditions. No interaction was found between contrast type and salience level (*F*(1,17) = 2.54; *p* = 0*.*13; η*_p_*^2^ = 0.13). We also compared the luminance-only and combined color/luminance conditions using paired *t*-tests (2_L_ against 2_L_/3_C_, 3_L_ against 3_L_/2_C_, 3_L_ against 3_L_/3_C_, 2_L_/3_C_ against 3_L_/3_C_, and 3_L_/2_C_ against 3_L_/3_C_). The PCN latency of the 2_L_/3_C_ condition was significantly faster than that of the 2_L_ condition (*t*(17) = –3.69; *p* = 0*.*002). No other comparisons were significant for latencies (all *t* < 1.27; all *p* > 0.22) or amplitudes (all *t* < 01.74; all *p* > 0.10).

### Discussion

In the second experiment, color and luminance were isolated or combined across two levels of salience to determine the effects on ERPs and behavior. The luminance-only and color-only conditions confirmed the findings from Experiment 1: Luminance-driven salience produced three difference wave components (i.e., early PCN, PCP, and late PCN), but color-driven salience produced a single component (i.e., PCN). Also, although higher salience resulted in higher amplitudes for both color and luminance PCN, only luminance had faster late PCN latencies for the higher salience. The EEG results were consistent with the behavioral results, in that higher salience conditions had faster RTs and higher accuracies compared to lower salience conditions. Likewise, the RTs of the luminance conditions were significantly faster than the RTs of the color conditions. Combining luminance and color signals resulted in a switch from the single color PCN to the three difference wave components, as in luminance-only waves. Although luminance and combination conditions had equivalent early PCN and PCP components, the 2_L_/3_C_ late PCN latency was faster than that of the 2_L_ condition. This faster PCN latency for the combination condition is mirrored in the RTs and accuracies, which were faster and higher, respectively, for 2_L_/3_C_ compared to 2_L_. No differences in late PCN latency and amplitude were found between 3_L_ and 3_L_/2_C_ or between 3_L_ and 3_L_/3_C_. A potential explanation for this lack of differences is the “winner-take-all” model of Zhaoping and Snowden (2006). If 3_L_ had higher salience than both 2_C_ and 3_C_, this would result in equivalent patterns of activity for 3_L_, 3_L_/2_C_, and 3_L_/3_C_ conditions, as the stronger contrast type (luminance) would determine the pattern of activity provoked.

No differences were found among the three combination conditions. This may be a result of saturation of the system. In other words, from a neuronal activation perspective, the combination neurons achieve maximum activation (limited by the number of neurotransmitter receptors). Another possible explanation is that the 2_C_ and 3_C_ conditions were not sufficiently different in salience to produce differences in PCN latency; although a difference in PCN amplitude was found between the two conditions, there was no significant difference between their PCN latencies.

Although significant differences were found between the RTs of the combination conditions and between the combination conditions and their equivalent luminance-only conditions, the differences were only 12, 11, 7, and 18 ms for 2_L_/3_C_ and 3_L_/3_C_, 3_L_/2_C_ and 3_L_/3_C_, 3_L_ and 3_L_/2_C_, and 3_L_ and 3_L_/3_C_, respectively. These RT differences are equal to or slightly smaller than the RT differences between high and medium (14 ms) and medium and low (18 ms) salience conditions in Töllner, Zehetleitner, Gramann, et al. (2011). This may have contributed to the lack of differences seen in PCN latency for the three combination conditions. The RT differences are consistent with the hypothesis that the salience of the combination conditions was dependent on both color and luminance, as higher salience of both features resulted in higher overall salience compared to higher salience of one feature and compared to stimuli with only one feature (whether higher or lower salience).

### General discussion

Previous EEG studies have explored attentional salience of different hues, but to our knowledge this study is the first to fully explore the effect of low-level color or luminance mechanisms on attentional salience and to use ERPs to determine how combinations of color and luminance affect various stages of attentional processing. We systematically varied color and luminance using a visual pop-out search for a higher contrast target to assess the degree to which the salience-computing attentional mechanisms are constrained by low-level visual inputs. In our first experiment, stimuli were defined by contrast that isolated chromatic or luminance mechanisms. In our second experiment, targets were defined by contrasts that isolated or combined achromatic and chromatic mechanisms. In both experiments, ERP waveforms contra- and ipsilateral to the target were qualitatively different for chromatic compared to luminance-defined stimuli. The same was true of the difference waves computed from these waveforms. The color-only difference waves reflected latency shifts between contra- and ipsilateral activity, and the luminance-only and combined color/luminance difference waves reflected amplitude shifts such as those observed in previous literature (Eimer & Kiss, 2008; Hopf, Luck, Girelli, Hagner, Mangun, Scheich, & Heinze, 2000; Töllner, Zehetleitner, Gramann, et al., 2011; Töllner et al., 2012; Töllner et al., 2015). Further, salience processing in the presence of luminance contrast differences elicited an early-onset, multicomponent selection process when compared to color, which elicited a single, more temporally constrained response. Our study is the first to provide conclusive evidence that early processing of attentional salience is sustained by distinct achromatic and chromatic mechanisms, which combine nonlinearly to drive attentional salience.

Hickey, Di Lollo, and McDonald (2009) reported results from a luminance-defined and an isoluminant stimulus, but the results for their isoluminant stimulus appear very similar to our luminance condition. This should not be surprising if we consider that their isoluminant stimulus was a line, thus allowing for edge artifacts, and that their isoluminance adjustments were performed only four times, which, with the very low overall background luminance used in their study, can result in a substantial residual luminance contrast. Therefore, it is our belief that we are the first to compare luminance-driven difference waves with truly isoluminant color-driven difference waves in this type of experimental paradigm. As in Hickey et al. (2009), luminance stimuli evoked multiple difference wave components. Wascher and Beste (2010) suggested that the N1pc is associated with an “initial orientation of attention” and the PCN with an “attention re-allocation,” likely representing a periodic sampling process in which the PCN is only elicited when people have missed the target in the first (N1pc) stage. The occurrence of the N1pc in the N1 window makes it somewhat similar to our early PCN. As achromatic RTs were faster than chromatic RTs, it is likely the difference wave component that allows distinction of target from distractors is earlier for achromatic compared to chromatic stimuli: the early PCN. Thus, the achromatic late PCN cannot be due to missing the target during the N1pc stage.

According to Eimer and Kiss (2008), the PCN (N2pc) only appears in the presence of a task-relevant target visual feature. They demonstrated this by presenting an uninformative color cue (a red singleton with five gray distractors) followed by a visual search task. The task involved either detection of a smaller gray bar among larger gray distractors and discrimination of its orientation or detection of a red bar among gray distractors and discrimination of its orientation. The cue only evoked a PCN when participants were asked to detect a red bar among gray distractors—that is, when the red feature of the uninformative cue was also a relevant feature in the visual search task. This implies that the late PCN is influenced by top-down processing and is involved in the filtering of targets from distractors. Perhaps instead, then, the early PCN can be said to reflect a bottom-up-driven initial allocation of perceptual resources and the late PCN to reflect additional target selection subprocesses of attentional allocation such as top-down-influenced target/non-target filtering of visual information. Additional filtering is not always required for target detection, as the target may be distinguished at the early PCN stage; however, it is possible for target detection to *not* occur purely through the bottom-up-driven early PCN, such as, for example, when the target and distractors are similar in salience.

At this point the target would be distinguished through top-down influences on pre-cortical neuronal networks that filter the visual information to suppress distractors and select for designated targets (the PCN). As the PCP occurs during the contralateral/ipsilateral post-N1 return to baseline, it is possible it also reflects subprocesses associated with the filtering of targets from distractors. However, as there are many subprocesses involved, it is not possible to determine whether the PCP and PCNs reflect the same, different, or multiple overlapping subprocesses from this study. Further experiments that isolate these various target/non-target filtering subprocesses would be needed to convincingly establish which of them the PCP and the early and late PCN are involved in.

As the late PCN is more closely tied to top-down influences, it is possible that salience computations it indexes reflect a stage that relates more to appearance (i.e., cortical color-opponent color representations) than to subcortical cone-opponent content. This might have been reasonably expected from a cortically generated difference wave, with sources in extrastriate areas (Hopf et al., 2000). Combining color with luminance resulted in difference wave patterns equivalent to those of achromatic stimuli, suggesting that luminance has a major impact on the latency and amplitude of ERPs when present in the stimulus. This indicates that summation of color and luminance is nonlinear. Such a result is in line with the “winner-take-all” model of Zhaoping and Snowden (2006), as the dominant EEG envelope is the one associated with the luminance signals, which have an early-onset, temporally distinct sequence of attentional difference wave components. However, this hypothesis is limited by the small number of color/luminance combination levels used in the study. Using these data, it is impossible to determine whether the transformation from the single PCN to the early PCN/PCP/late PCN difference wave pattern is sudden or gradual with the addition of luminance signal. These two behaviors would point to the “winner-take-all” model and a nonlinear additive mode, respectively. A further experiment with a finer gradation of color/luminance combinations would be required to determine which is the case. Faster PCN latency when color was added to a less salient level of luminance contrast (2_L_/3_C_) also suggests either a nonlinear summation of the two low-level salience signals or activation of combination color/luminance neurons. Thus, although luminance appears to be the driving force behind the early PCN and PCP difference wave components, when the luminance signal is less salient the late PCN appears to be influenced by both luminance and chromaticity contrast. Note that the present findings bear close resemblance with previous ERP studies exploring the locus of the redundant-signals effect (e.g., Töllner, Zehetleitner, Krummenacher, & Müller, 2011; Krummenacher, Grubert, Töllner, & Müller, 2014). Similarly, these authors observed enhanced PCN amplitudes for targets redundantly defined by two feature dimensions (color and orientation) relative to when the target was defined by just one feature dimension (color or orientation). This pattern of effects was taken to provide further support for the notion that the PCN can be regarded as a neural measure associated with attentional selection at the level of the attention-guiding master (i.e., saliency or priority) map. Recent findings from perceptual research are also consistent with these findings. Participants perceived checkerboard patterns that stimulated double-opponent cells (i.e., cells sensitive to both color and luminance) as more saturated than uniform squares that stimulated single-opponent cells (sensitive to color only) even when the stimuli were equated in space-averaged cone contrast (Nunez, Shapley, & Gordon, 2018).

Our results are also in line with the attentional selection study by Martinovic and Andersen (2018). They found that steady-state visual evoked potentials produced by luminance and color combination stimuli could not be predicted using the steady-state visual evoked potentials produced by luminance-isolating and color-isolating stimuli, indicating that the cortical response to a combination of luminance and color cannot be explained by summation of the cortical responses to the two individually. Thus, the nonlinear processing of combined luminance and color signals must be taken into consideration by attention researchers who study the neural mechanisms of attentional selection using color stimuli that are not isoluminant with the background.

Putting all together, it is likely that the selection of targets that differ from distractors in luminance contrast involves temporally distinct subprocesses of attentional target selection: (1) the early PCN (initial allocation of perceptual resources) and (2) the late PCN (top-down-influenced target selection). By contrast, color targets seem to rely on either a single process or a temporally concurrent combination of processes that enable distinction of the target from its surrounding distractors. Neural mechanisms that compute attentional salience are thus constrained by the low-level inputs they receive, revealing that the processing of stimuli that include both color and luminance contrast (such as those we would normally encounter in everyday life) is largely driven by sites that combine the two types of signals in a nonlinear fashion.

## Supplementary Material

Supplement 1
