# Supplementary material for: Neural differences between chromatic- and luminance-driven attentional salience in visual search
Source: J Vis. 2020 Mar 20;20(3):5. doi: 10.1167/jovi.20.3.5 (PMC7408945; doi:10.1167/jovi.20.3.5)
Supplement: Supplement 1 [file jovi-20-3-5_s001.docx]

Supplementary Materials: Salience Matching Experiment


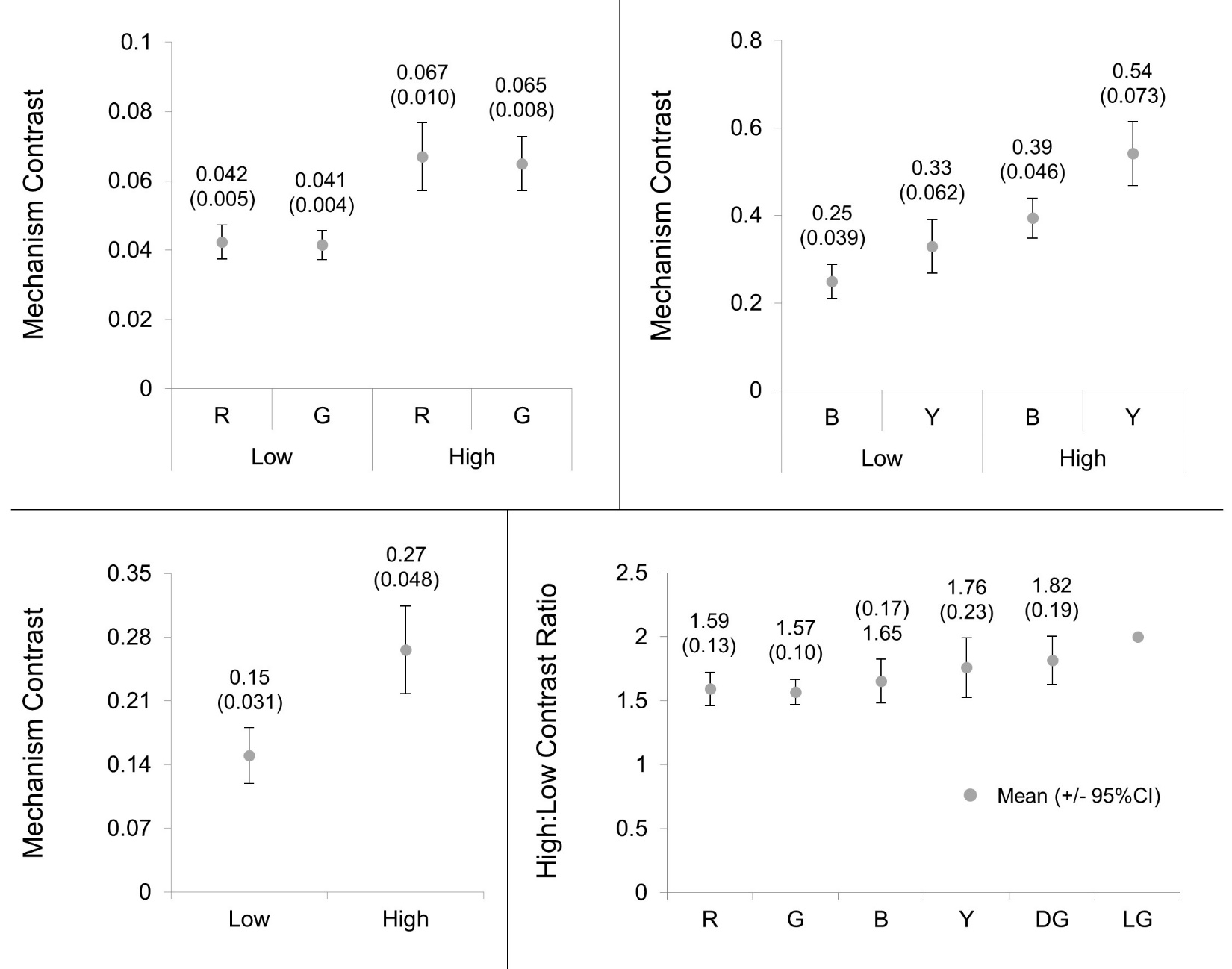


*Figure 1* – Results from the salience adjustment task, showing mean adjustments for low and high contrast stimuli for a) the reddish (R)/greenish (G) mechanism b) the bluish (B)/yellowish (Y) mechanism c) the dark grey (DG) from the light grey (LG)/DG mechanism and d) the mean high:low contrast ratios from all six conditions (including the LG standard ratio of 2 as a reference). The means and 95% confidence intervals (in brackets) are above each data point. The results are shown in terms of R/G, B/Y and LG/ DG mechanism contrasts and mean contrast ratios, respectively.

First, we assessed if salience was similar between the two poles of the same cone-opponent mechanism. Two 2x2 repeated measures ANOVAs (with factors of salience level and color direction) were performed for the reddish/greenish and bluish/yellowish adjustment results. High salience levels had significantly higher contrasts than low salience levels for both reddish/greenish (*F*(1,18)=82.3, *p*<.001, η_p_^2^=.82) and bluish/yellowish (*F*(1,18)=98.3, *p*<.001, η_p_^2^=.85). Yellowish had higher mean contrasts than bluish (*F*(1,18)=33.2, *p*<.001, η_p_^2^=.65). No effect of color direction was found for reddish/greenish (*F*(1,18)=1.02, *p*=.33, η_p_^2^=.054), nor was an interaction found (*F*(1,18)=0.24, *p*=.63, η_p_^2^=.013). On the other hand, an interaction between salience level and color direction was found for bluish/yellowish (*F*(1,18)=15.8, *p*=.001, η_p_^2^=.47). Paired *t*-tests showed yellowish had higher mean contrasts at both low (*t*(18)=3.88, *p*=.001) and high salience (*t*(18)=6.62, *p*<.001). Calculations of Cohen’s *d* showed the effect size was larger for high salience (*d*=1.88) than low salience (*d*=1.04), indicating that the interaction was likely to be driven by the larger difference at high salience. A paired *t*-test between salience levels of dark grey found high salience dark grey to have a significantly larger mean contrast than low salience dark grey (*t*(18)=8.03, *p*<.001). One-sample *t*-tests comparing the high and low dark grey results to the light grey standard stimuli showed the dark grey contrast was significantly lower than the standard at both high (*t*(18)=-4.91, *p*<.001) and low salience (*t*(18)=-2.78, *p*=.012).

To determine whether the contrast ratios between high and low matches were similar for the two chromatic mechanisms, a 2x2 repeated measures ANOVA (with factors of color mechanism and color direction) was performed. There was no significant effect of mechanism (*F*(1,18)=2.75, *p*=.12, η_p_^2^=.13) or direction (*F*(1,18)=0.37, *p*=.55, η_p_^2^=.020), nor was there an interaction between the two factors (*F*(1,18)=3.10, *p*=.095, η_p_^2^=.15). Paired-sample *t*-tests between the four chromatic ratios and the dark grey ratios (Bonferroni corrected *p*=.013) showed the dark grey ratio was not significantly different from any of the chromatic ratios: reddish (*t*(18)=2.75, *p*=.013), greenish (*t*(18)=2.61, *p*=.018), bluish (*t*(18)=1.74, *p*=.10), yellowish (*t*(18)=0.55, *p*=.59). One-sample *t*-tests between the five experimentally derived ratios and the fixed ratio of 2 used for the standard stimulus (Bonferroni corrected *p*=.010) showed the ratios were significantly lower than the fixed ratio for reddish (*t*(18)=-6.09, *p*<.001), greenish (*t*(18)=-8.49, *p*<.001) and bluish (*t*(18)=-3.97, *p*=.001), but not for yellowish (*t*(18)=-2.05, *p*=.056) or dark grey (*t*(18)=-1.92, *p*=.071).

Discussion

Switkes (2008) found a trend of <1 reddish:greenish ratio and a >1 bluish:yellowish ratio at equal salience. Here, participants chose contrasts that were approximately equal for the reddish/greenish mechanism and were larger for yellowish than bluish. The differences in results may have been due to a number of factors, including differences in stimulus (large gratings in Switkes, small Gaussians in our experiment), paradigm (2AFC in Switkes, adjustment in our experiment) and individual differences between participants (Switkes had 4 experienced observers, while we had a large and mixed group). Although an experiment into the effects of spatial stimulus attributes and tasks on salience matching found that the differences in results were probably due to the difference in stimuli rather than task, these results are not the main finding of the experiment, they will not be discussed further (Hardman and Martinovic 2017).

Supplementary Materials: Between-Subject Variation in ERPs


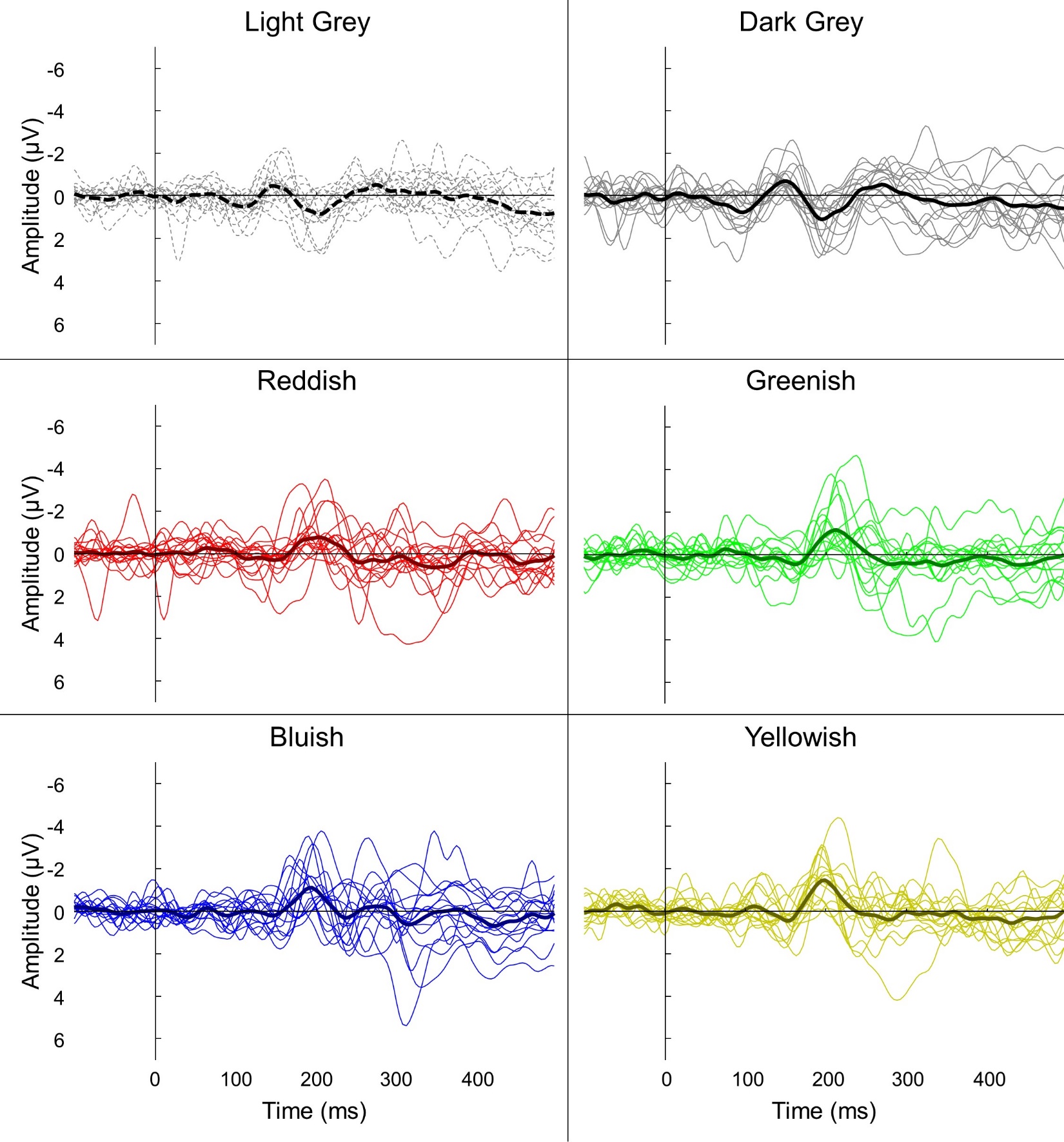


*Figure 2* – Difference waveforms for the six conditions of Experiment 1. Each plot contains the grand-means (thick line) and individual participants’ ERPs (thin lines). Note the more scattered activity for reddish compared to the other conditions.

From the graphs of *Figure 2*, it is obvious that the reddish condition contained larger variation in ERPs between individuals compared to the other conditions. Thus, when taking the average, the maximum activity was lower than would be expected from averaging the maximum activity of all participants (as occurred in the statistics used).

References

Hardman, A. and Martinovic, J. (2017, August). *Salience of spatiochromatic patterns measured with adjustment and alternative forced choice tasks*.  Poster session presented at the 24^th^ Symposium of the International Colour Vision Society, Erlangen, Germany.

Switkes, E. (2008). Contrast salience across three-dimensional chromoluminance space. *Vision Research*. 48, 1812-1819.
